# Comparative Transcriptome Analysis Reveals Genes Associated with the Gossypol Synthesis and Gland Morphogenesis in *Gossypium hirsutum*

**DOI:** 10.3390/genes13081452

**Published:** 2022-08-15

**Authors:** Cuiping Zhang, Xiuyan Liu, Yin Song, Zhengran Sun, Jinli Zhang, Hao Wu, Yuzhen Yang, Zhenkai Wang, Daohua He

**Affiliations:** College of Agronomy, Northwest A&F University, No. 3 Taicheng Road, Xianyang 712100, China

**Keywords:** *Gossypium hirsutum*, transcriptome analysis, gossypol, glands

## Abstract

*Gossypium hirsutum* is an important source of natural textile fibers. Gossypol, which is a sesquiterpenoid compound mainly existing in the cotton pigment glands, can facilitate resistance to the stress from diseases and pests. The level of gossypol in the cotton is positively correlated to the quantity of pigment glands. However, the underlying regulatory mechanisms of gossypol synthesis and gland morphogenesis are still poorly understood, especially from a transcriptional perspective. The transcripts of young leaves and ovules at 30 DPA of the glanded plants and glandless plants were studied by RNA-Seq and 865 million clean reads were obtained. A total of 34,426 differentially expressed genes (DEGs) were identified through comparative transcriptome analysis. Genes related to gossypol synthesis or gland morphogenesis displayed significant differential expression between the two cultivars. Functional annotation revealed that the candidate genes related to catalytic activity, the biosynthesis of secondary metabolites, and biomolecular decomposition processes. Our work herein unveiled several potential candidate genes related to gossypol synthesis or gland morphogenesis and may provide useful clues for a breeding program of cotton cultivars with low cottonseed gossypol contents.

## 1. Introduction

Cotton is a critical economic crop, since it not only provides high-quality natural fibers, but its seeds are also rich in natural nutrients such as oil, protein, and mineral elements [1]. The protein exported from cottonseed globally could potentially meet the basic protein needs of 500 million people a year [2]. Pigment glands and gossypol are crucial traits of cotton, and they will affect the growth and development of cotton. Cotton plants lacking pigment glands and gossypol are vulnerable to pests and diseases, which will result in lower yield [3]. Pigment glands are distributed throughout the various organs of cotton plants including the roots, stems, leaves, seeds, etc., in which gossypol is mainly stored [4,5,6]. Gossypol, as a sesquiterpene compound, can resist the invasion of pests and diseases and enhance the adaptability of plants to unfavorable environments [7]. However, gossypol is highly toxic to humans and monogastric animals, which directly limits the full utilization of cottonseed by humans and causes a lot of waste of resources [8].

Cadinene synthase genes (*CADs*) are key genes in the process of gossypol synthesis and can be divided into two subfamilies: A subfamily (*cad1-A*) and C subfamily (*cad1-C1*, *cad1-C2*, *cad1-C3*, *cad1-C4* and *cad1-C14*) [9,10,11]. As the seeds mature, the transcription level of the *cad1* gene increases sharply and the content of gossypol in the seeds accumulates rapidly. However, the content of sesquiterpenes such as gossypol was greatly reduced by interfering with the expression of this gene [9,10,11,12]. In 2006, Sunilkumar et al. employed RNAi technology along with a seed-specific promoter to specifically express the dsRNA of the *CAD* gene in seeds to inhibit the expression of this gene in seeds, thereby reducing the content of gossypol in the cottonseeds while the presence of gossypol in the other parts remained intact [2,13]. Wagner et al. blocked the synthesis of gossypol by interfering with the expression of the cytochrome gene (*P450*), which greatly reduced the gossypol content [14]. 3-Hydroxy-3-methylglutaryl CoA reductase (HMGR) is the first rate-limiting enzyme in the gossypol synthesis pathway. Loguercio et al. cloned two *HMGR* genes, *hmg1* and *hmg2*, from *G. hirsutum* and found that the expression of these two genes in a short period of time was consistent with the morphogenesis of cotton pigment glands and the synthesis of gossypol [15]. It was also found that *hmg2* was related to the morphogenesis of pigment glands and the synthesis of sesquiterpenes by using external mechanical damage or pathogen invasion [15]. In addition, some studies discovered that isopentenyl diphosphate isomerase (IPI) and farnesyl diphosphate synthase (FPS) are also vital enzymes in the gossypol biosynthetic pathway [16,17]. There is a complicated relationship between cotton pigment glands and gossypol. Generally, cotton with pigmented glands contains a large amount of terpenoids [7], but in contrast, there is a very low gossypol content in glandless cotton. The cavities of the pigment glands are developed by special cells through lysis. The original special cells of the pigment glands are surrounded by flat cells. As the cell walls of special cells dissolve to form a cavity, the flat secretory cells inside the cavity slowly degrade. The final mature pigment gland structure is a cavity surrounded by one layer of secretory cells and one to three layers of flat and degenerated sheath cells [18]. Studies have shown that the formation of pigment glands in *G. hirsutum* leaves was a programmed cell death (PCD) event that was regulated by plant development [18]. The development of pigment glands in *G. hirsutum* shows many autophagy and characteristics of PCD mediated by autolysis such as the collapse of cell walls, chromosome aggregation, nuclear membrane disintegration and nuclear degradation, autolysate structure surrounded by membranes, etc. [18]. In 1998, Zhu et al. conducted a study on the tissue structure of seeds and cotyledons of cotton with the characteristics of the delayed formation of cotyledon pigment glands [19]. He inferred that the genes that control the delayed formation of pigment glands in Australian wild cotton seeds are actually certain enzymes or enzyme activators that control the disintegration of pigment gland cells [19]. Cotton cultivars with delayed formation of cotyledon pigment glands do not have enzymes or enzyme activators that disintegrate pigment glands during seed maturation. Therefore, the pigment gland cells in the pigment gland cannot be disintegrated into the pigment gland cavity. After the seeds germinate, the enzymes are synthesized or activated, and the pigment gland cells in the pigment glands are quickly disintegrated to form the pigment gland cavity [19]. Then, pigment substances such as gossypol are imported into the glands to gradually form visible pigmented glands.

In 1954, McMichael identified the first pigment gland formation gene *gl_1_* through genetic analysis. This recessive gene mainly controls the formation of pigment glands in cotyledons and leaves [20]. Subsequently, they discovered another five gene loci (*gl_2_*, *gl_3_*, *gl_4_*, *gl_5_*, *gl_6_*) related to the glandless traits of cotton and found that each gene loci had multiple alleles such as *Gl_2_^e^*. Madhusudhana et al. cloned *Gl_2_^e^* and overexpressed the gene, which led to a sharp increase in the content of gossypol and related terpenoids. At the same time, the gene was knocked out by CRISPR/CAS9 technology, resulting in the appearance of a glandless phenotype [21].

Since the morphogenesis of glands and the synthesis of gossypol involve complex biological processes, the research on its potential regulatory mechanism has not been perfected. At the same time, compared with gossypol synthesis, we know little about the genes engaged in gland morphogenesis. In addition, the molecular mechanism of gland formation is still unclear. However, the prerequisite for cultivating cotton plants with gossypol and gossypol-free seeds is to explore the gene network related to gland morphogenesis and gossypol synthesis. Although previous studies have found that specific genes and pathways can reduce the gossypol content, the transcriptome network of glandular morphogenesis and gossypol synthesis deserves further study.

## 2. Materials and Methods

### 2.1. Materials

Tetraploid *G. hirsutum* (86 III 72 glanded and 86 III 72 glandless) were planted in the greenhouse of Northwest A&F University in Yangling, China. All tissues of 86 III 72 glanded cotton plants were covered with glands, and a large amount of gossypol was stored in the glands. There were no glands in any tissue of the 86 III 72 glandless cotton plants.

### 2.2. RNA Sampling and Extraction

The cotton (86 III 72 glanded and 86 III 72 glandless) bolls of 30 day post-anthesis (DPA) and young leaves were taken for study. The cotton bolls were cut with a sterile scalpel, and the ovules were peeled off before it was quickly put into liquid nitrogen to extract the RNA. Three individual biological replications were included to minimize the experimental error. According to the manufacturer’s instructions (Tiangen Biochemical Technology (Beijing, China) Co., Ltd.), the total RNA was extracted from all of the samples using the RNA prep Pure Plant Kit. The concentration and purity of the extracted RNAs were measured using agar gel electrophoresis and a Nanodrop micro spectrophotometer (Thermo Scientific, Waltham, MA, USA).

### 2.3. Sequence Quality Control and Read Mapping

Illumina’s NEBNext^®^ Ultra^TM^ RNA Library Prep Kit was used to construct the library. Quality control checks for all of the libraries were performed with an Agilent 2100 Bioanalyzer system according to the manufacturer’s protocols. The qualified cDNA libraries were ultimately sequenced by Illumina and 150 bp paired-end reads were generated. The original data were filtered to ensure the quality and reliability of the data analysis. It mainly included removing reads with adapters, removing reads containing N (N means that the base information cannot be determined), and removing low-quality reads (reads whose base number of Qphred ≤ 20 accounted for more than 50% of the entire read length). At the same time, the Q20, Q30, and GC content of the clean data were calculated. HISAT2 v2.0.5 was employed to construct the index of the reference genome, and HISAT2 v2.0.5 was used to map clean reads to the reference genome [22]. The featureCounts tool in the subread software was used to calculate the reads mapped to each gene [23]. The gene expression level was normalized by using the FPKM method. FPKM refers to the expected number of fragments per kilobase of transcript sequence fragments sequenced per million base pairs. The Pearson correlation coefficients of samples within and between groups were calculated based on the FPKM value of all genes in each sample. In addition, principal component analysis (PCA) on the gene expression value (FPKM) of all samples was performed by the PCA provided by the Limma package [24].

### 2.4. Analysis of DEGs and Functional Annotation

DESeq2 software (1.16.1) was utilized to analyze the differential expression between the two comparison combinations. |log2(FoldChange)| ≥1 and FDR ≤ 0.05 were used as the thresholds for significant differential expression. Enrichment analysis of DEGs was realized by clusterProfiler R software. All differentially expressed genes were annotated to Gene Ontology (GO) and the Kyoto Encyclopedia of Genes and Genomes (KEGG) pathways [25,26]. The GO term or pathway with FDR ≤ 0.05 was regarded as the GO term or pathway with significant enrichment of differentially expressed genes. The local version of the gene set enrichment analysis (GSEA) tool (http://www.broadinstitute.org/gsea/index.jsp, accessed on 8 September 2021) was used to perform GSEA analysis on the GO, KEGG, and other datasets of this species [27].

### 2.5. Transcription Factors (TFs) Analysis of DEGs

The online website PlantTFDB [28] (Plant Transcription Factor Database) 4.0 (http://planttfdb.cbi.pku.edu.cn/, accessed on 20 September 2021) was used to identify the differentially expressed transcription factors in cotton.

### 2.6. Weighted Gene Co-Expression Network Analysis

The R package WGCNA was used to describe the gene association patterns between the different samples. This algorithm was based on high-throughput mRNA expression data [29,30]. In the WGCNA analysis, the weighted value of the correlation coefficient was adopted, so the connections between genes in the network obeyed the scale-free network distribution. We set the threshold of the square of the correlation coefficient to 0.8, and the weight parameter (i.e., soft threshold) β = 12. According to the determined soft threshold, the network was constructed, and the dissimilarity between genes was used to hierarchically cluster the genes in the network and build a hierarchical clustering tree. The tree was cut into different modules (the minimum number of genes in the module was 30) by the dynamic shearing method. The modules whose correlation coefficient was greater than 0.75 (i.e., the dissimilarity coefficient was less than 0.25) were merged. Cytoscape software version 3.6.0 (Seattle, WA, USA) [31] was used to visualize the co-expression networks.

### 2.7. Protein Interaction Network Analysis

The protein interaction network analysis of the gene set was based on the STRING database. Diamond (0.9.13) was used to align the target gene sequences with the *Gossypium raimondii* protein sequence. The protein interaction network was established according to the known protein interaction relationships in *G. raimondii*. The protein interaction network diagram was visually edited by Cytoscape [31].

### 2.8. Analysis of Alternative Splicing

Alternative splicing is an indispensable mechanism for regulating the gene expression and protein variables. Alternative splicing (AS) events were analyzed by rMATS (3.2.5) software (Iowa, IA, USA) [32] including skipped exon (SE), mutually exclusive exon (XE), alternative 5′ splice site (A5SS), alternative 3′ splice site (A3SS), and retained intron (RI).

### 2.9. SNP/InDel Annotations

Tools such as picard-tools were used to sort the chromosome coordinates and remove duplicate reads from the comparison results of the variant sites. SNP Calling and Indel Calling variant sites were analyzed by GATK [33]. The SNP/InDel of the glanded cotton and the glandless cotton relative to the reference genome were obtained after filtering the original results. The common SNP/InDel was filtered out to obtain the SNP/InDel between the glanded cotton and the glandless cotton. Finally, ANNOVAR was used for SNP/InDel annotation [34], and statistical analysis of the annotation information of variant sites was performed according to the SnpEff software (Detroit, MI, USA [35]).

### 2.10. qRT-PCR Analysis

The cotton (86 III 72 glanded and 86 III 72 glandless) ovules of 30 DPA and young leaves were collected, and total RNA were extracted using an RNAprep Pure Kit (Tiangen Biochemical Technology (Beijing, China) Co., Ltd.). Each sample included three individual biological replicates. cDNA was synthesized using HiScript^®^Q RT SuperMix for qPCR (Vazyme, China) according to the protocol for qRT-PCR analysis. The qRT-PCR primers of genes were designed through Primer Premier 5.0 (Table S11). The qRT-PCR was carried out in a 20 uL volume, with three replicates for each sample including 10 μL 2 × SuperReal Color PreMix, each of the forward and reverse primers 0.6 μL (10 μM), 1 μL cDNA (50 ng μL^–1^), 0.4 μL 50 × ROX reference dye, and 7.4 μL RNase-free ddH_2_O. The qRT-PCR reaction conditions were 95 °C for 15 min, followed by 40 cycles of 95 °C for 10 s and 60 °C for 30 s in the PCR stage. Data acquisition and analyses were performed using the QuantStudio^TM^ Real-Time PCR Software (Thermo Fisher Scientific, Waltham, MA, USA). The data were normalized by the internal control gene *UBQ7* (*DQ116441*) and the relative expression level was calculated using the 2^–ΔΔCT^ analysis method [36].

## 3. Results

### 3.1. Comparison of the Phenotypic Characteristics of Glanded Cotton and Glandless Cotton

**Figure 1:**
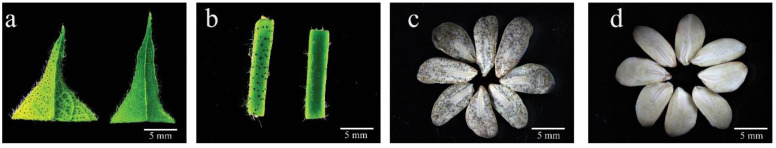
The phenotypic investigation of different tissues (leaves, stems, seeds) of the glanded cotton and glandless cotton. (**a**) The young leaves of glanded cotton (**left**) and glandless cotton (**right**). (**b**) The stems of glanded cotton (**left**) and glandless cotton (**right**). (**c**) The seeds of the glanded cotton. (**d**) The seeds of the glandless cotton.

**Figure 2:**
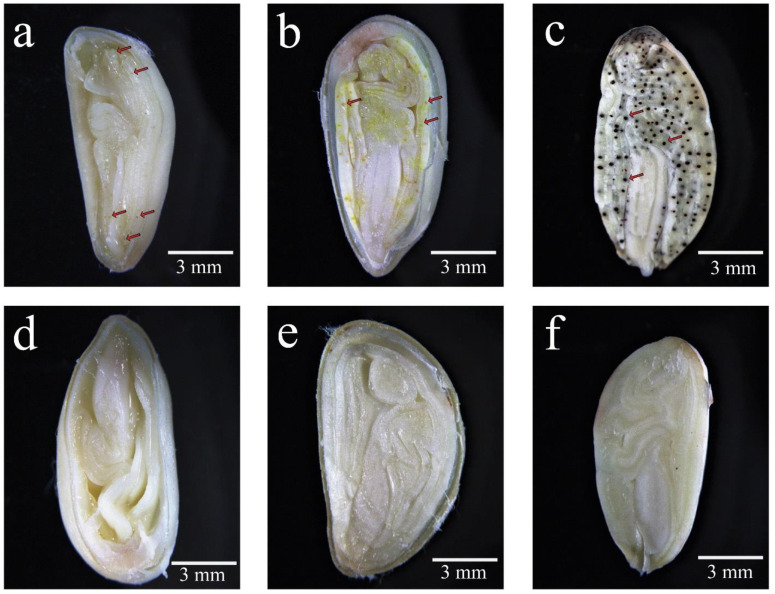
The phenotypic investigation of ovules of the glanded cotton and glandless cotton at different stages. (**a**–**c**) 20 DPA, 30 DPA and mature seeds of glanded cotton, respectively. (**d**–**f**) The 20 DPA, 30 DPA, and mature seeds of glandless cotton, respectively. The red arrow points to the gland.

To understand the phenotypes of glands in different tissues and cotton accessions, the condition of the glands in their young leaves, stems, and seeds were investigated. There were a large number of pigment glands in the leaves, stems, and seeds of the glanded cotton while there were no pigment glands in any tissue of the glandless cotton (Figure 1a–d). In addition, we also compared the glands in the 20 DPA, 30 DPA ovules, and mature seeds of the glanded cotton and glandless cotton. A small amount of tiny pigment glands could be seen in the ovules in the 20 DPA of glanded cotton (Figure 2a). A large number of pigment glands could be observed in the 30 DPA ovules of glanded cotton (Figure 2b), and its volume was much larger than 20 DPA. Comparing the 30 DPA ovules and the mature seeds of the glanded cotton, the mature seeds were significantly darker in color (Figure 2c). This may be caused by the low content of phenolic compounds in the gland cavity in the 30 DPA ovules. However, we did not observe glands in the 20 DPA, 30 DPA, and mature seeds of the glandless cotton (Figure 2d–f).

### 3.2. Transcriptome Sequencing, Assembly, and Annotation

High-throughput RNA-Seq generated 923 million raw reads from 12 RNA samples using the Illumina HiSeq^TM^ 2000 platform, which was approximately 77 million reads for each sample. After discarding the adapters, poly-N and low quality reads from raw data, a total of 865 (93.71%) million clean reads were obtained. Overall, 90.82% (87.44–92.11%) of the high-quality reads were mapped to the *G. hirsutum* reference genome [37] using HISAT2 v2.0.5. Of the mapped reads, 79.17% (76.27–83.10%) were aligned with exon regions, while 2.03% (0.97–2.67%) were aligned with introns and 18.80% (15.93–21.37%) were mapped onto intergenic regions (Table S2). The average Q20, Q30, and GC contents were 97.72% (96.76–98.11%), 93.42% (91.09–94.29%), and 43.96% (43.54–44.54%), respectively. The error rate of all samples was 0.03% (Table S1). The mapped data were displayed as the following three categories: (1) multiple mapped (4.65–6.33%) and unique mapped reads (87.44–92.11%); (2) forward mapped (43.71–46.04%) and reverse mapped reads (43.74–46.06%); and (3) non-splice reads (53.32–59.52%) and splice reads (32.24–35.444%) (Table S2). In the comparison with the *G. hirsutum* reference genome, 44,799 known genes and 2280 novel genes were identified. In summary, RNA-Seq provided abundant information, which facilitated exploring reliable transcription and expression clues. Considering that the RNA-Seq samples differed in pigment gland morphogenesis, the RNA-Seq data provided the evidence and clues to explore the genes underlying pigment gland morphogenesis and gossypol synthesis from the perspectives of gene differential expression and DNA polymorphism in the transcription region.

### 3.3. Correlation Analysis of Samples

In order to reveal the repeatability between samples and validate the correctness of sequencing, we performed correlation analysis and principal component analysis (PCA) on the gene expression quantity of each sample. The data showed that the Pearson correlation coefficients between the biological replicates were greater than 0.8 (0.818 < R^2^ < 0.973) (Figure S1A). Additionally, PCA revealed four distinctive clusters (Figure S1B), which implied that the biological replicates from the same group were highly correlated. In summary, the results of the principal component analysis (PCA) of the twelve samples were consistent with the results from correlation analysis.

### 3.4. Identification of DEGs between Glanded Cotton and Glandless Cotton

**Figure 3:**
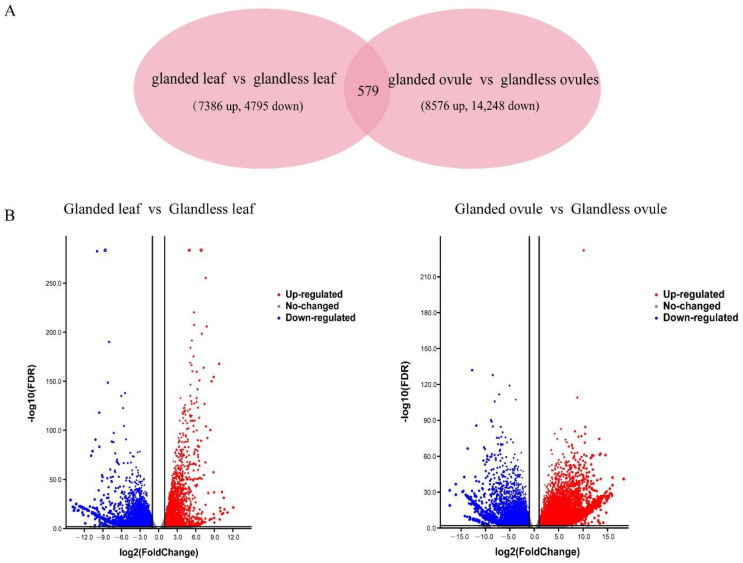
The total number of DEGs in two groups. (**A**) Venn diagram of DEGs in glanded cotton and glandless cotton (the leaves of glanded cotton vs. glandless cotton and the ovules of glanded cotton vs. glandless cotton). The numbers in each circle represent the number of DEGs in the corresponding group. The overlapped part of the circle represents the common DEGs between the groups. (**B**) Volcano plots of the glanded cotton and glandless cotton in the two groups. The *x*-axis represents log base two-fold change, the *y*-axis represents –logbase 10 FDR for each plot. The upregulated DEGs are indicated by red dots; the downregulated DEGs are indicated by blue dots and non-DEGs are indicated by gray dots.

The differential expression of genes results in divergent phenotypic character controlled by specific biological pathways. Using DESeq2 software, we identified 12,760 DEGs in the leaf comparison of glanded cotton and glandless cotton (7635 genes were upregulated and 5125 genes were downregulated; Figure 3A,B). A total of 23,403 DEGs were observed in the ovules in the comparison of glanded cotton and glandless cotton (8826 were upregulated and 14,577 were downregulated; Figure 3A,B). Of these, only 579 DEGs were specifically expressed in the two comparisons (Figure 3A). The 579 DEGs were very likely to be involved in gossypol synthesis or gland morphogenesis.

### 3.5. The DEGs of Gossypol Biosynthetic Pathway

**Figure 4:**
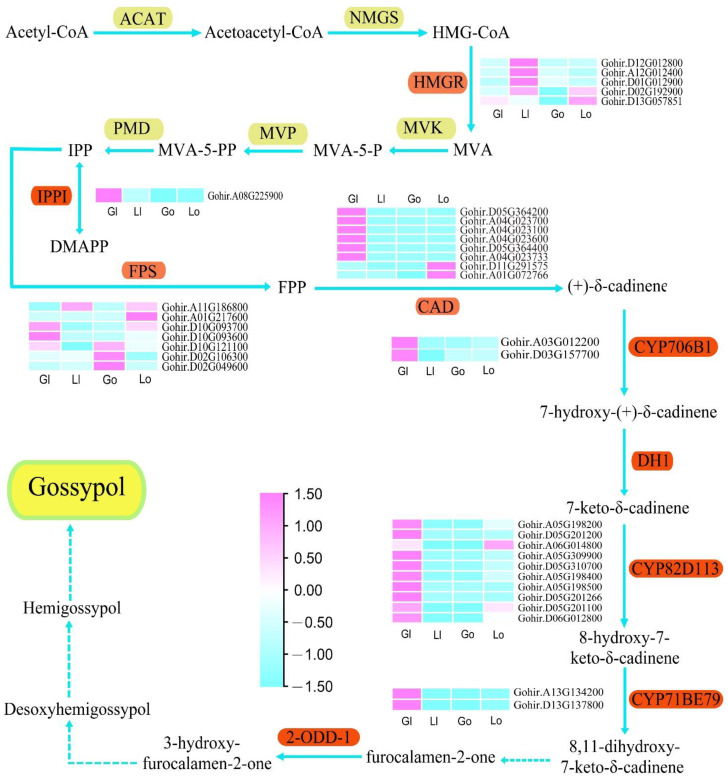
The expression patterns of the key genes in the gossypol synthesis pathway. Data were obtained using the log_2_FPKM of each gene. From top to bottom in the figure are *HMGR*, *IPPI*, *FPS*, *CAD*, *CYP706B1*, *CYP82D113*, and *CYP71BE79*. Gl, Ll, Go, and Lo represent the leaves of the glanded cotton, leaves of glandless cotton, ovules of glanded cotton, and ovules of glandless cotton, respectively. Dashed arrows indicate unidentified reaction(s). (Pathway from [6]).

Gossypol is mainly synthesized through the mevalonate (MVA) pathway, which involves several key enzymes including 3-hydroxy-3-methylglutaryl-CoA (HMGR), isopentenyl diphosphate isomerase (IPPI), farnesyl diphosphate synthase (FPS), cadinene synthase (CAD), and plant cytochrome (P450) [6,15,16,17,38]. In order to reveal the regulatory relationship of genes in the gossypol synthesis pathway, we investigated the expression patterns of the above genes (Figure 4). The three *HMGR* genes (*Gohir.D12G012800*, *Gohir.A12G012400*, *Gohir.D01G012900*) showed similar expression patterns and were strongly expressed in the ovules of glanded cotton. *Gohir.D02G192900* and *Gohir.D13G057851* were abundantly expressed in the glanded cotton and glandless cotton. The IPPI gene (*Gohir.A08G225900*) was highly expressed in the leaves of glanded cotton, but not expressed in glandless cotton. The seven *FPS* genes had various expression patterns: *Gohir.D10G093600* was highly expressed in the leaves of glanded cotton, while *Gohir.D02G106300* and *Gohir.D02G049600* were highly expressed in the leaves of the glandless cotton. Moreover, *Gohir.A01G217600* were strongly expressed in the ovules of glandless cotton, and the rest of the genes were highly expressed in both the glanded cotton and glandless cotton. Interestingly, eight *CAD* genes showed diametrically opposite expression patterns, *Gohir.D11G291575* and *Gohir.A01G072766* were only expressed in the ovules of glandless cotton, and the remaining six *CAD* genes were only expressed in the leaves of glanded cotton. In particular, Zhang reported that silencing *CAD* gene *Ghi_A04G01066* by VIGS technology reduced the number, size, and color intensity of the pigment glands. Our sequence alignment indicated that *Ghi_A04G01066* is probably the same as *Gohir.A04G023700* [39]. In addition, we also found that *P450* (*CYP706B1*, *CYP82D113* and *CYP71BE79*) genes are more likely to be highly expressed in the leaves of glanded cotton such as *Gohir.A03G012200*, *Gohir.D03G157700,* and *Gohir.A13G134200*, etc.

### 3.6. TFs Involved in Gossypol Synthesis or Gland Morphogenesis

**Figure 5:**
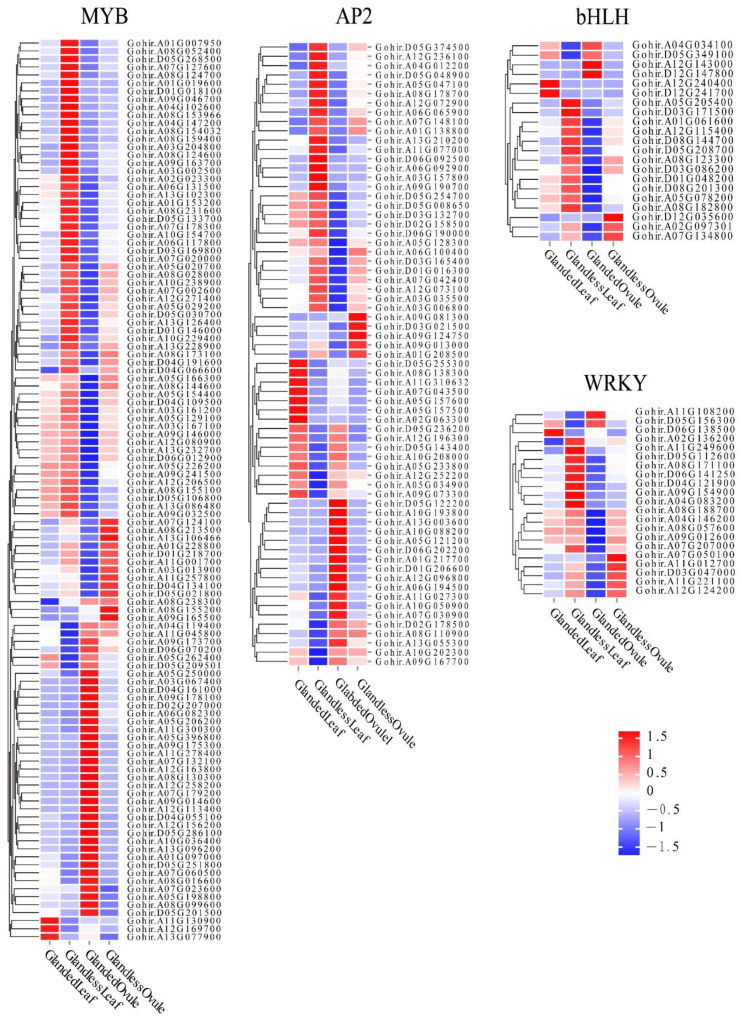
The heat map of the TF gene expression levels.

Previous studies have shown that gossypol synthesis or gland morphogenesis was regulated by several transcription factors. To identify TFs associated with gossypol synthesis or gland morphogenesis, we studied the expression patterns of *WRKY*, *MYB*, *bHLH*, and *AP2*. In total, 750 differentially expressed TF genes were identified in the comparison of glanded cotton and glandless cotton. The most abundant differentially expressed TF gene was *MYB* (319), followed by *AP2* (249), *WRKY* (140) and *bHLH* (42). TF genes that were highly expressed in the leaves (or ovules) of glanded cotton and lowly expressed in the leaves (or ovules) of glandless cotton or the opposite expression patterns were deeply analyzed. These genes may be critical in gossypol synthesis or gland morphogenesis (Figure 5).

### 3.7. DEGs GO/Pathway Enrichment Analysis

**Figure 6:**
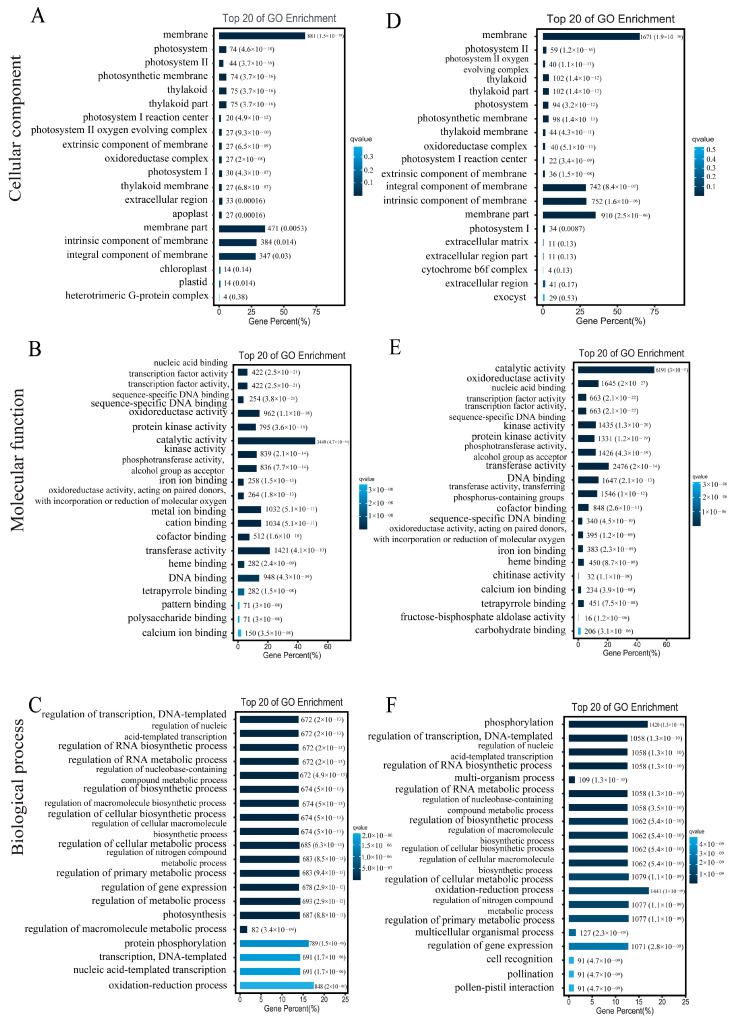
The GO enrichment analysis of the DEGs in different tissues of glanded cotton and glandless cotton. (**A**–**C**) Classification of the enriched GO terms in the leaves of glanded cotton and glandless cotton. (**D**–**F**) Classification of the enriched GO terms in the ovules of glanded cotton and glandless cotton.

To explore the biological functions of the DEGs, we performed GO annotation analysis. The 12,760 DEGs in the comparison between the leaves of the glanded cotton and glandless cotton were mainly enriched in several different terms such as membrane (G0:0016020), catalytic activity (GO:0003824), etc. (Figure 6A–C). The 23,403 DEGs in the comparison of ovules of the glanded cotton and glandless cotton were enriched in catalytic activity (GO:0003824), membrane (GO:0016020), etc. (Figure 6D–F).

**Figure 7:**
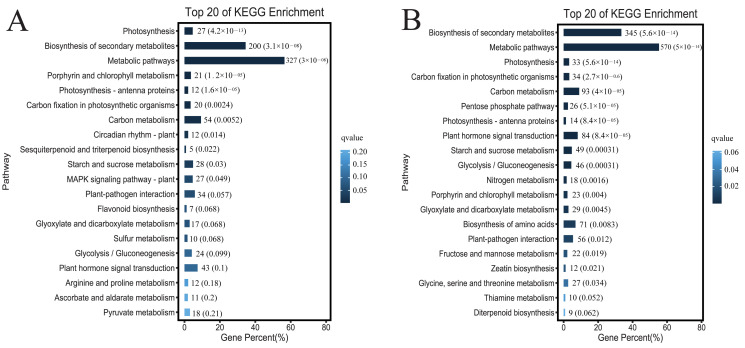
The KEGG pathway analysis of the DEGs in different tissues of the glanded cotton and glandless cotton. (**A**) Top 20 of the KEGG analysis in the leaves of glanded cotton and glandless cotton. (**B**) Top 20 of the KEGG analysis in the ovules of the glanded cotton and glandless cotton.

To explore which biological metabolic pathways the DGEs participate in, we conducted an enrichment analysis of the KEGG pathway. The DEGs in the comparison between the leaves of the glanded cotton and glandless cotton were significantly enriched in the biosynthesis of secondary metabolites (ko01110) and metabolic pathways (ko01100) (Figure 7A). The DEGs in the comparison between the ovules of the glanded cotton and glandless cotton were remarkably enriched in the biosynthesis of secondary metabolites (ko01110) and metabolic pathways (ko01100) (Figure 7B).

The above GO annotation and KEGG pathway analysis discovered that these DEGs were mainly enriched in the catalytic activity (catalytic activity (GO:0003824)) and metabolic process (biosynthesis of secondary metabolites (ko01110)). The results also correspond to the fact that gossypol is a secondary metabolite, and the morphogenesis of glands requires a large number of enzymes to catalyze the degradation of pigment gland cells.

In order to more accurately find genes related to gossypol synthesis or gland morphogenesis, we analyzed 579 DEGs that existed in two comparison groups (leaves of glanded cotton vs. glandless cotton, ovules of glanded cotton vs. glandless cotton). These genes included cytochrome monooxygenase genes (PF00067: Cytochrome P450) (*Gohir.A02G114800*, *Gohir.A07G079400*, *Gohir.A10G235200*), *AP2* transcription factors (PF00847: AP2 domain) (*Gohir.A06G065900*, *Gohir.D05G236200*), and *Gohir.D01G034700* (PF00179: Ubiquitin-conjugating enzyme), and *Gohir.D10G218500* (PF00544: Pectate lyase), (Table S3). In addition, we performed GO enrichment analysis and pathway analysis on these 579 genes. The results showed that these genes were significantly enriched in the cytoplasmic part (GO:0044444), intracellular part (GO:0044424), and ribosome (ko03010) (Table S4). These genes were differentially expressed in the leaves of the glanded cotton and glandless cotton, and they were also differentially expressed in the ovules of the glanded cotton and glandless cotton. Therefore, we speculate that the 579 DEGs are closely related to gossypol synthesis or gland morphogenesis.

### 3.8. GSEA Analysis

In order to avoid missing some genes that were not significantly differentially expressed but had important biological significance, we conducted a GSEA. We removed gene sets with fewer than 15 and more than 5000 genes and a total of 80,255 genes were involved in GSEA analysis.

In the comparison with the leaves of the glanded cotton and glandless cotton, the results showed that the genes highly expressed in the leaves of the glanded cotton were significantly enriched in peptide N-acetyltransferase activity (GO:0034212), exocytosis (GO:0006887), secretion (GO:0046903), plant–pathogen interaction (ATH04626), autophagy-other (ATH04136), other glycan degradation (ATH00511), the mapk signaling pathway-plant (ATH04016), and endocytosis (ATH04144) (Table S5). Genes that were highly expressed in the leaves of the glandless plants were hugely enriched in intramolecular lyase activity (GO:0016872), aldehyde-lyase activity (GO: 0016832), and circadian rhythm-plant (ATH04712) (Table S6).

In the comparison of ovules of the glanded cotton and glandless cotton, the results indicated that genes remarkably expressed in the ovules of glanded cotton were greatly enriched for the regulation of actin polymerization or depolymerization (GO:0008064), phagosome (ATH04145), endocytosis (ATH04145), and endocytosis (ATH04145) (Table S7). Genes extremely expressed in the ovules of glandless plants were considerably enriched in plant–pathogen interaction (ATH04626), plant hormone signal transduction (ATH04075), and the mapk signaling pathway-plant (ATH04016) (Table S8).

### 3.9. Protein Interaction Network Analysis

**Figure 8:**
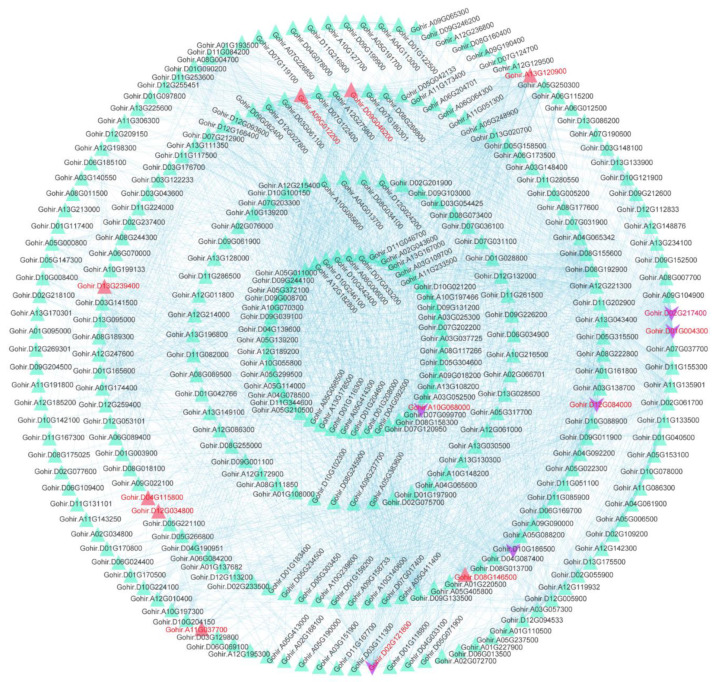
The protein interaction network of important gene sets in the GSEA analysis. The purple arrows represent hub genes. The pink triangles represent hub genes that may be involved in apoptosis.

The GSEA analysis revealed that DEGs in the cotton of glanded cotton and glandless cotton were relevant to a variety of biomolecular decomposition processes. We performed a protein interaction network analysis on 1575 genes enriched in key gene sets of the GSEA analysis results (e.g., exocytosis (GO:0006887) and autophagy-other (ATH04136), etc.) (Figure 8). Based on the weights of these genes, we further studied the hub genes (Table S9).

The results showed that the hub genes in the interaction network were *Gohir.D02G217400* and *Gohir.D01G004300*. The annotation results of these two genes were bifunctional dihydrofolate reductase-thymidylate synthase (PF00186: Dihydrofolate reductase and PF00303: Thymidylate synthase). The interaction network also included the hub genes *Gohir.D10G186500*, *Gohir.D12G084000*, *Gohir.D02G121800*, and *Gohir.A10G068000*.

In addition, we found that some hub genes may be related to apoptosis. These genes mainly included *Gohir.A09G012200* (PF10408: Ubiquitin elongating factor core), *Gohir.D12G034800* (PF00632: HECT-domain (ubiquitin-transferase)), *Gohir.D06G160100* (PF00240: Ubiquitin family), *Gohir.D08G146500* (PF04111: Autophagy protein Apg6), *Gohir.A11G037700* (PF00179: Ubiquitin-conjugating enzyme), *Gohir.D13G239400* (PF16190: Ubiquitin-activating enzyme E1 FCCH domain; PF09358: Ubiquitin fold domain), and *Gohir.D11G338300* (PF00240: Ubiquitin family). The expression levels of these genes in the glanded cotton plants were slightly higher than those in the glandless cotton. We speculate that these hub genes involved in apoptosis may be related to the morphogenesis of glands.

### 3.10. Weighted Gene Co-Expression Network Analysis

**Figure 9:**
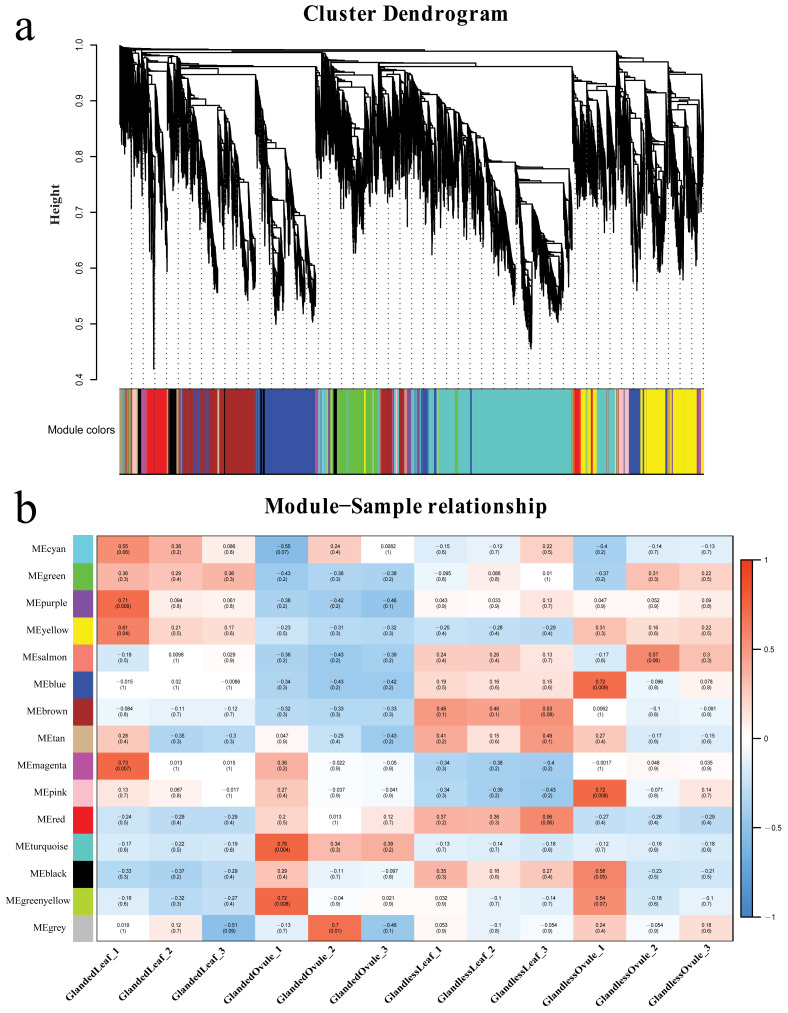
The WGCNA of the transcripts in the glanded cotton and glandless cotton. (**a**) Cluster dendrogram of different genes in the coexpression modules. (**b**) The relationships between co-expressed modules and different samples in the glanded cotton and glandless cotton.

To understand the regulatory network of genes associated with gossypol synthesis and cotton gland morphogenesis, a weighted gene co-expression network analysis was performed on 12 samples and their expression datasets (42,091 genes in total). We performed hierarchical clustering of genes and built a hierarchical clustering tree (Figure 9a). In the end, we identified a total of 14 modules and the number of genes in each module was between 112 and 6371. Different modules were marked with different colors, and 20 genes that did not belong to any module were placed in the gray module. Four modules (cyan, green, purple, yellow) were extremely positively correlated with the leaves of glanded cotton; four modules (red, turquoise, black, green, yellow) were negatively correlated with the leaves of glanded cotton; six modules (green, purple, yellow, salmon, blue, brown) were negatively correlated with the ovules of glanded cotton; four modules (salmon, blue, brown, tan) were significantly positively correlated with the leaves of glandless cotton (Figure 9b).

We found that the gene expression patterns in the four modules (salmon, blue, brown, magenta) were significantly correlated with the phenotypes of different samples. For example, three of the modules (salmon, blue, brown) were negatively related to the leaves and ovules of the glanded cotton, but positively related to the leaves and ovules of the glandless cotton. In contrast, one module (magenta) was positively correlated with the leaves and ovules of glanded cotton, but negatively correlated with the leaves and ovules of glandless cotton. We hypothesize that the genes in the salmon module, blue module, brown module, and magenta module may be related to the synthesis of gossypol or the morphogenesis of glands, so we conducted more in-depth research on these four modules.

To explore the function of the genes in these four modules, we conducted enrichment analysis (GO and Pathway enrichment analysis) for the genes in each module. The analytical results showed that the 135 genes of the salmon module were enriched in GO terms such as apoplast (GO:0048046), extracellular region (GO:0005576), circadian rhythm-plant (ko04712), and plant–pathogen interaction (ko04626) (Figure S2). The blue module had a total of 3490 genes, which were significantly enriched in GO terms such as translation (GO:0006412), peptide metabolic process (GO:0006518), ribosome (ko03010), and ubiquinone and other terpenoid-quinone biosynthesis (ko00130) (Figure S3). The brown module had 2877 genes and they were enriched in the biosynthetic process (GO:0009058), terpenoid backbone biosynthesis (ko00900), and fatty acid metabolism (ko01212) (Figure S4). There were 479 genes in the magenta module, which were enriched in proteolysis involved in the cellular protein catabolic process (GO:0051603), protein catabolic process (GO: 0030163), organonitrogen compound catabolic process (GO:1901565), cellular macromolecule catabolic process (GO:0044265), organic substance catabolic process (GO:1901575), proteasome (ko03050), and ubiquitin mediated proteolysis (ko04120) (Figure S5). The genes of these four modules were all enriched in processes related to the metabolism of proteins and other biomolecules. In addition, the genes of the magenta module were mainly enriched in the catabolism process of biomolecules.

**Figure 10:**
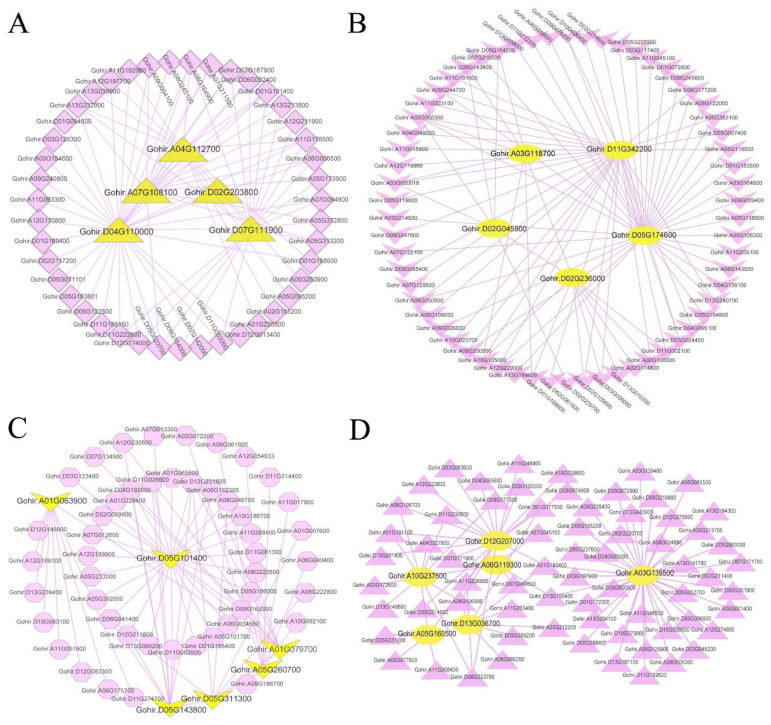
The coexpression network of the hub genes in four modules. (**A**) Salmon module. (**B**) Blue module. (**C**) Magenta module. (**D**) Brown module. The yellow represents hub genes.

To find the key genes related to gossypol synthesis or gland morphogenesis, we analyzed the gene co-expression network of these four modules separately and selected the genes with higher weights in each module to analyze and draw the network (Table S10). In the salmon module, we found several hub genes (Figure 10A) including *Gohir.D04G110000* (PF00722: Glycosyl hydrolases family 16) and *Gohir.A07G108100* (PF04949: Transcriptional activator), etc. In the blue module (Figure 10B), the observed hub genes included *Gohir.D02G236000* (PF08041: PetM family of cytochrome b6f complex subunit 7), *Gohir.A03G118700* (PF10716: NADH dehydrogenase transmembrane subunit), *Gohir.D11G342200* (PF00067: Cytochrome P450), etc. In the brown module (Figure 10D), the hub genes included *Gohir.A10G237600* (PF00487: Fatty acid desaturase), *Gohir.D13G036700* (PF00067: Cytochrome P450), *Gohir.A06G119300* (PF00875: DNA photolyase; PF03441: FAD binding domain of DNA photolyase), etc. In the magenta module (Figure 10C), the hub genes included *Gohir.A05G260700* (PF00067: Cytochrome P450), *Gohir.A01G079700* (PF00069: Protein kinase domain), *Gohir.A01G063900* (PF00179: Ubiquitin-conjugating enzyme), etc.

In the gene co-expression network, the hub genes play a central role in the network because they interact with a large number of genes. According to our analytical results, it can be speculated that the hub genes *Gohir.D04G110000* (PF00722: Glycosyl hydrolases family 16) and *Gohir.A01G063900* (PF00179: Ubiquitin-conjugating enzyme) may play a key role in the process of apoptosis and promote the morphogenesis of glands. Because the cytochrome monooxygenase gene (PF00067: Cytochrome P450) is an important gene in the process of gossypol synthesis, the three hub genes *Gohir.D11G342200*, *Gohir.A05G260700,* and *Gohir.D13G036700* may be of great significance in the process of gossypol synthesis.

### 3.11. Validate RNA-Seq by qRT-PCR Assays

**Figure 11:**
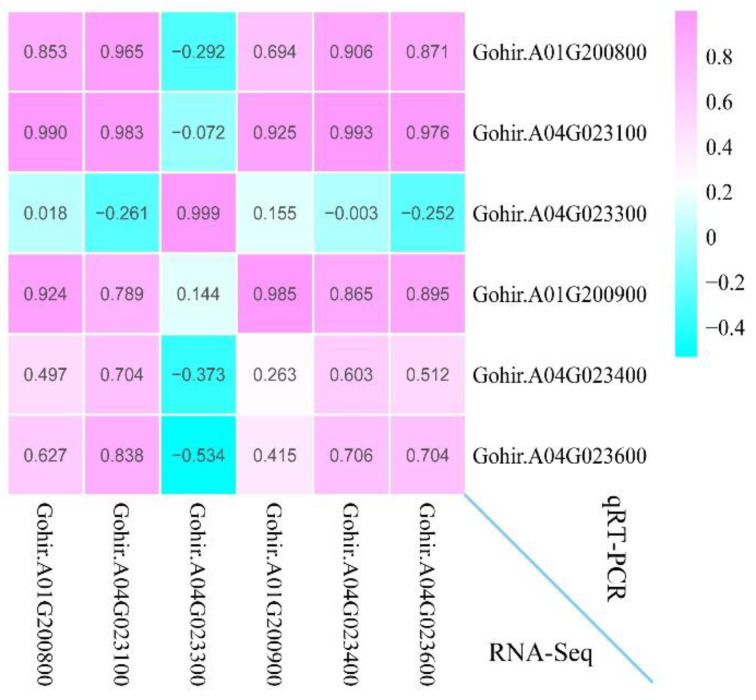
The correlation between the RNA-Seq data (FPKM) and qRT-PCR results (2^−ΔΔCT^) of the six genes. The numbers in the grid represent the Pearson correlation coefficient.

To validate the accuracy of RNA-Seq, the relative expression level of six randomly selected genes was examined using qRT-PCR analysis (Figure 11). The data showed that the correlation coefficients of the relative expression level and FPKM were all greater than 0.6 (0.603 < R^2^ < 0.999). These results support the reliability of our RNA-Seq and subsequent interpretations.

### 3.12. Alternative Splicing Analysis

Alternative splicing is an important mechanism for regulating gene expression. For the same gene, there were different splicing methods between the glanded cotton and glandless cotton, which may be related to the presence or absence of glands and the synthesis of gossypol. Through rMATS (3.2.5) software analysis, it was found that in the comparison of the leaves of glanded cotton and glandless cotton, the number of skipped exon (SE) was the largest, with 15,634. The second was the alternative 3′ splice site (A3SS), with a total of 3075. In the comparison of plant ovules of the glanded cotton and glandless cotton, the most variable splicing was still SE with 17,282, followed by A3SS with 2983 (Table S12).

To find important alternatively spliced genes, we investigated differentially significant alternative splicing (FDR < 0.05) (Table S12). We found that most genes had only one type of alternative splicing, but some genes had more than one such as *Gohir.A05G341300*, which had A3SS and SE. In addition, we also found that the same alternative splicing occurred at different sites on some genes. For example, there were two sites on *Gohir.A03G150000* with SE alternative splicing and three sites in *Gohir.A12G044100* with SE alternative splicing. Among these genes with differentially significant alternative splicing, we observed that some genes such as *Gohir.A02G039300*, *Gohir.A03G034300*, *Gohir.A03G037300*, *Gohir.A04G109800*, and *Gohir.A07G188500* were alternatively spliced in both the leaves (glanded cotton vs. glandless cotton) and ovules (glanded cotton vs. glandless cotton). Furthermore, some genes had the same type of alternative splicing in the leaves (glanded cotton vs. glandless cotton) and ovules (glanded cotton vs. glandless cotton) such as *Gohir.D01G123500*, which had A3SS in both the leaves (glanded cotton vs. glandless cotton) and ovules (glanded cotton vs. glandless cotton). We speculate that these genes with the same type of alternative splicing in both leaves (glanded cotton vs. glandless cotton) and ovules (glanded cotton vs. glandless cotton) play important roles in gossypol synthesis or gland morphogenesis. We also performed alternative splicing analysis on candidate genes and found that one AP2 (*Gohir.A12G072900*) and four hub genes (*Gohir.A06G040400*, *Gohir.D13G190400*, *Gohir.D12G231600*, *Gohir.D06G041400*) had alternative splicing in the glanded cotton and glandless cotton. In addition, we found that some genes may be related to apoptosis in these genes with differentially significant alternative splicing such as *Gohir.A01G114000* (PF00179: Ubiquitin-conjugating enzyme), *Gohir.A02G078600* (PF00443: Ubiquitin carboxyl-terminal hydrolase), and *Gohir.D02G090500* (PF00179: Ubiquitin-conjugating enzyme). In summary, we believe that these variable splicing between glanded cotton and glandless cotton may be one of the reasons for the presence or absence of glands.

### 3.13. Analysis of Variant Sites

Based on the comparison of the RNA-Seq data with the reference genome, we identified the SNPs and InDels of the transcriptional segment of the glanded cotton and glandless cotton. As a result, 2167 SNPs and 184 InDels were found and these variant sites were annotated (Table S13). A total of 64.84% (1405/2167) of SNPs and 21.19% (39/184) of InDels appeared in the protein coding region. The main reason for the high polymorphism of the protein coding region was that the mining of polymorphism was based on the RNA-Seq data, and only the polymorphism of the ‘transcribable segment’ in the genome was studied. A total of 35.16% (762/2167) of SNPs and 78.80% (145/184) of InDels appeared in the intergenic region. The variant annotation also showed that eight InDels and three SNPs had a high impact (high). Between the glanded cotton and glandless cotton, InDels caused frameshift mutations in six genes (*Gohir.A01G207600*, *Gohir.A12G154200*, *Gohir.A12G160000*, *Gohir.D02G026300*, *Gohir.D05G374400*, *Gohir.D08G072200*), and SNPs led to the gain or loss or alternative splicing of the terminator of three genes (*Gohir.A12G161800*, *Gohir.A12G175966*, *Gohir.D13G226500*). The SNPs and InDels that existed between the glanded cotton and glandless cotton had a high impact as well as the genes in which they were located, which may have been the cause of the presence or absence of glands.

## 4. Discussion

Nowadays, there is limited understanding of the molecular mechanism of cotton gland morphogenesis and gossypol synthesis as well as the precise regulatory network. In this study, we performed transcriptome sequencing on young leaves and 30 DPA ovules of glanded cotton and glandless cotton. The DEGs between the two varieties were analyzed. A total of 12,760 DEGs were identified in the leaves of the glanded cotton and glandless cotton. Then, 23,403 DEGs were identified in the ovules of the glanded cotton and glandless cotton. The DEGs in the ovules of the glanded cotton and glandless cotton were far more than the DEGs in the leaves of the glanded cotton and glandless cotton. The possible reason was that the morphogenesis time of the glands in the ovules of the glanded cotton started gradually around 16 DPA [21], while the young leaves of the glanded cotton already had a large number of glands. In addition, there were more genes downregulated than upregulated in the ovules of the glanded cotton, which may be involved in a large number of negative regulations in the process of gossypol synthesis or gland morphogenesis. We speculate that in the process of gossypol synthesis (accumulation), the synthesized gossypol (or gossypol precursor) may be converted into other substances in a high amount, which ultimately leads to a decrease in the accumulation of gossypol in glandless plants.

A large number of PCD was mediated by autophagy and autolysis appeared during the development of the pigment glands of *G. hirsutum* such as cell wall collapse, chromosome aggregation, nuclear membrane disintegration, and nuclear degradation. The cell wall was often degraded in the early stages of pigment gland development. This phenomenon is considered to be one of the functions of plant PCD [40,41]. In the central cells of the pigment glands, epithelial cells, and sheath cells, the cytoplasm was surrounded by irregular membranous structures. From the perspective of morphological structure, this membranous structure was similar to autolysates in the PCD process of animal and plant cells [42,43,44]. Studies have shown that this membranous autolysate contains lytic enzymes, which can degrade the contents of cells including various organelles [45]. Membranous autolysates or cytoplasm fuse with vacuoles and enter the vacuoles. At the same time, a large number of flocculent substances and autolysate structures appear in the vacuoles. Therefore, we infer that the degradation of the cytoplasm of the pigment glands of *G. hirsutum* may be caused by autophagosomes and vacuole-mediated autophagy.

In our study, a combination of GO analysis, Pathway analysis, GSEA analysis, and WGCNA analysis showed that cotton gland morphogenesis involved a large number of genes related to apoptosis. GSEA detects gene sets rather than individual gene expression changes, so it can contain subtle expression changes. In the process of GSEA analysis, we found that genes highly expressed in the leaves and ovules of glanded cotton were significantly enriched in processes related to programmed cell death and biological processes related to actin such as exocytosis (GO:0006887), secretion by cell (GO:0032940), terpene synthase activity (GO:0010333), exocyst (GO:0000145), endocytosis (ATH04144), fatty acid degradation (ATH00071), autophagy-other (ATH00 0008154), actin cytoskeleton organization (GO:0030036), endocytosis (ATH04144), ubiquitin mediated proteolysis (ATH04120), and phagosome (ATH04145). In addition, we identified several hub genes related to apoptosis such as *Gohir.D09G046200*, *Gohir.D12G034800*, *Gohir.D06G160100*, *Gohir.A13G120900*, etc. WGCNA analysis revealed that there were four modules that may be related to the synthesis of gossypol or the morphogenesis of glands. GO and Pathway analyses of the genes in these four modules indicated that these genes were involved in a large number of catabolic processes and processes related to cell apoptosis such as cellular protein catabolic process (GO:0044257), organic substance catabolic process (GO:1901575), fatty acid metabolism (ko01212), ubiquitin mediated proteolysis (ko04120), proteasome (ko03050), ubiquinone and other terpenoid-quinone biosynthesis (ko00130), etc. Some hub genes were identified in the four modules such as *Gohir.A01G063900* (PF00179: Ubiquitin-conjugating enzyme), *Gohir.D11G342200* (PF00067: Cytochrome P450), *Gohir.A05G260700* (PF00067: Cytochrome P450), and these genes may play an important role in gossypol synthesis or gland morphogenesis.

It has been reported that transcription factors such as MYB, WRKY, bHLH, AP2, etc. may be associated with gossypol synthesis or gland morphogenesis, and genes such as *CAD* are closely connected to gossypol synthesis [15,46,47,48,49]. The MYB family is a multifunctional protein family that participates in the regulation of various physiological and biochemical processes in plants including controlling cell morphogenesis, regulating plant secondary metabolism, participating in plant hormone response, and plant resistance regulation and other aspects [50,51]. In 2020, Gao reported that phenotypic screening of the genes via virus-induced gene silencing showed an apparent disappearance of pigmented glands after the silencing of a pair of homologous MYB-encoding genes [52]. In the present study, the MYB transcription factor family was identified as the most abundant transcription factors differentially expressed in different cotton accessions. Of these, the expression levels of most *MYB* in the glandless cotton were higher than the glanded cotton and they had similar expression patterns. However, we found a small number of genes with unique expression patterns such as *Gohir.A13G077900*, *Gohir.A12G169700*, and *Gohir.A11G130900* were only highly expressed in leaves of the glanded cotton, and *Gohir.A09G165500* and *Gohir.A08G155200* were only highly expressed in the ovules of glandless cotton. The expression of *CAD1-A* is regulated through the binding of the transcription factor WRKY to the promoter of *CAD1-A*. The expression of *CAD1-A* is increased by the overexpression of *WRKY*. The expression of *WRKY* can be induced by *Verticillium dahliae*, which is faster than *CAD1-A* [47]. Our results identified one *WRKY* TF (*Gohir.D05G156300*), which was mainly expressed in the glanded cotton. Five *WRKY* TFs (*Gohir.A02G136200*, *Gohir.A12G124200*, *Gohir.A11G012700*, *Gohir.D03G047000,* and *Gohir.A11G221100*) were mainly expressed in the glandless cotton, and they may negatively regulate the expression of *CAD*. Afifi et al. revealed that the glandless trait was controlled by the multiple allele of *Gl_2_* and named it *Gl_2_^e^* [53]. Cheng HL found that *Gl_2_^e^* belonged to the bHLH-MYC transcription factor family. After analyzing the expression of *MYC*, it was found that the gene was expressed in the glanded cotton but not in the glandless cotton [54]. In this study, we discovered two *bHLH-MYC* (*Gohir.A12G143000* and *Gohir.D12G147800*) that were strongly expressed in the ovules of glanded cotton. The possible reason is that we sampled at 30 DPA, when the glands in the ovule were still in the morphogenesis stage. The AP2/ERF transcription factor family is one of the largest transcription factor families in the plant kingdom [55] and it can be induced to be expressed by ethylene [56]. Cotton glands are the result of programmed cell death [18], and ethylene plays a very important regulatory role in the process of programmed cell death. It is speculated that the AP2/ERF transcription factor may be involved in gland development. In our study, we found that three *AP2* (*Gohir.D05G236200*, *Gohir.A05G233800,* and *Gohir.D05G143400*) were highly expressed in cotton with glands, but not expressed in cotton without glands.

A large number of variable splicing and mutation sites (SNP and InDel) in two cotton varieties (glanded cotton and glandless cotton) were discovered through transcriptome sequencing and genome comparison. It was also found that the most frequent type of variable splicing was SE, followed by A3SS through variable splicing analysis. These alternative splicing may affect the formation of glands or the synthesis of gossypol. Gene expression will be affected by variant sites at different locations. Our analysis of variant sites showed that eight SNP sites and three InDel sites had a higher impact. Nine genes with 11 mutation sites may play an imperative role in the formation of cotton glands, and further verification is needed.

## 5. Conclusions

In summary, the genes that may be related to gossypol synthesis or gland morphogenesis were screened out through comparative transcriptome analysis. It was shown that these genes were involved in a large number of processes related to programmed cell death, which also proved that the formation of cotton glands was a result of cell apoptosis. The identified hub genes may pave the way to understanding the molecular mechanism of gossypol synthesis and gland morphogenesis. This study also provided a useful reference for further in-depth study of the molecular mechanisms of gossypol synthesis and gland morphogenesis as well as the precise regulatory networks.

## Data Availability

The transcriptome data of 86 III 72 glanded and 86 III 72 glandless cotton plants are available from the corresponding author (daohuahe@nwafu.edu.cn) upon reasonable request. The plant materials are available from the corresponding author upon request.

## Supplementary Materials

The following supporting information can be downloaded at: www.mdpi.com/article/10.3390/genes13081452/s1, Figure S1. The correlation analysis of 12 samples of glanded cotton and glandless cotton. (A) Pearson’s correlation coefficients of the sequencing data of 12 samples; (B) Principal component analysis (PCA) of the sequencing data of 12 samples. Figure S2. Enrichment analysis of genes in the salmon module. (A–C) Classification of the enriched GO terms in the salmon module. (D) Top 20 associated KEGG pathways for the salmon module. Figure S3. Enrichment analysis of genes in the blue module. (A–C) Classification of the enriched GO terms in the blue module. (D) Top 20 associated KEGG pathways for the blue module. Figure S4. Enrichment analysis of genes in the brown module. (A–C) Classification of the enriched GO terms in the brown module. (D) Top 20 associated KEGG pathways for the brown module. Figure S5. Enrichment analysis of genes in the magenta module. (A–C) Classification of the enriched GO terms in the magenta module. (D) Top 20 associated KEGG pathways for the magenta module. Table S1. The quality of transcriptome sequencing. Table S2. Gene mapping rate. Table S3. DEGs that exist both in the comparison of the glanded cotton and glandless cotton leaves and the comparison of glanded cotton and glandless cotton ovules; Table S4. GO analysis of 579 DEGs that existed in the two comparison groups (leaves of glanded cotton vs. glandless cotton, ovules of glanded cotton vs. glandless cotton); the KEGG analysis of 579 DEGs that existed in two comparison groups (leaves of glanded cotton vs. glandless cotton, ovules of glanded cotton vs. glandless cotton). Table S5. GSEA: GO analysis of genes that were highly expressed in the leaves of glanded cotton in the comparison of glanded cotton and glandless cotton leaves; GSEA: KEGG analysis of genes that were highly expressed in the leaves of the glanded cotton in the comparison of glanded cotton and glandless cotton leaves. Table S6. GSEA: GO analysis of genes that were highly expressed in the leaves of glandless cotton in the comparison of glanded cotton and glandless cotton leaves; GSEA: KEGG analysis of genes that were highly expressed in the leaves of glandless cotton in the comparison of the glanded cotton and glandless cotton leaves. Table S7. GSEA: GO analysis of genes that were highly expressed in the ovules of the glanded cotton in the comparison of the glanded cotton and glandless cotton ovules; GSEA: KEGG analysis of genes that are highly expressed in the ovules of the glanded cotton in the comparison of the glanded cotton and glandless cotton ovules. Table S8. GSEA: GO analysis of genes that were highly expressed in the ovules of glandless cotton in the comparison of glanded cotton and glandless cotton ovules; GSEA: KEGG analysis of genes that were highly expressed in the ovules of glandless cotton in the comparison of glanded cotton and glandless cotton ovules. Table S9. Hub genes in the important gene set in the GSES. Table S10. Hub genes in the salmon module; hub genes in the blue module; hub genes in the brown module; hub genes in the magenta modul. Table S11. List of the primers used for qRT-PCR. Table S12. Alternative splicing. Table S13. SNP annotation.
