# Supplementary figures and images for: Comparative Transcriptome Analysis Reveals Genes Associated with the Gossypol Synthesis and Gland Morphogenesis in Gossypium hirsutum
Source: Genes (Basel). 2022 Aug 15;13(8):1452. doi: 10.3390/genes13081452 (PMC9408450; doi:10.3390/genes13081452)

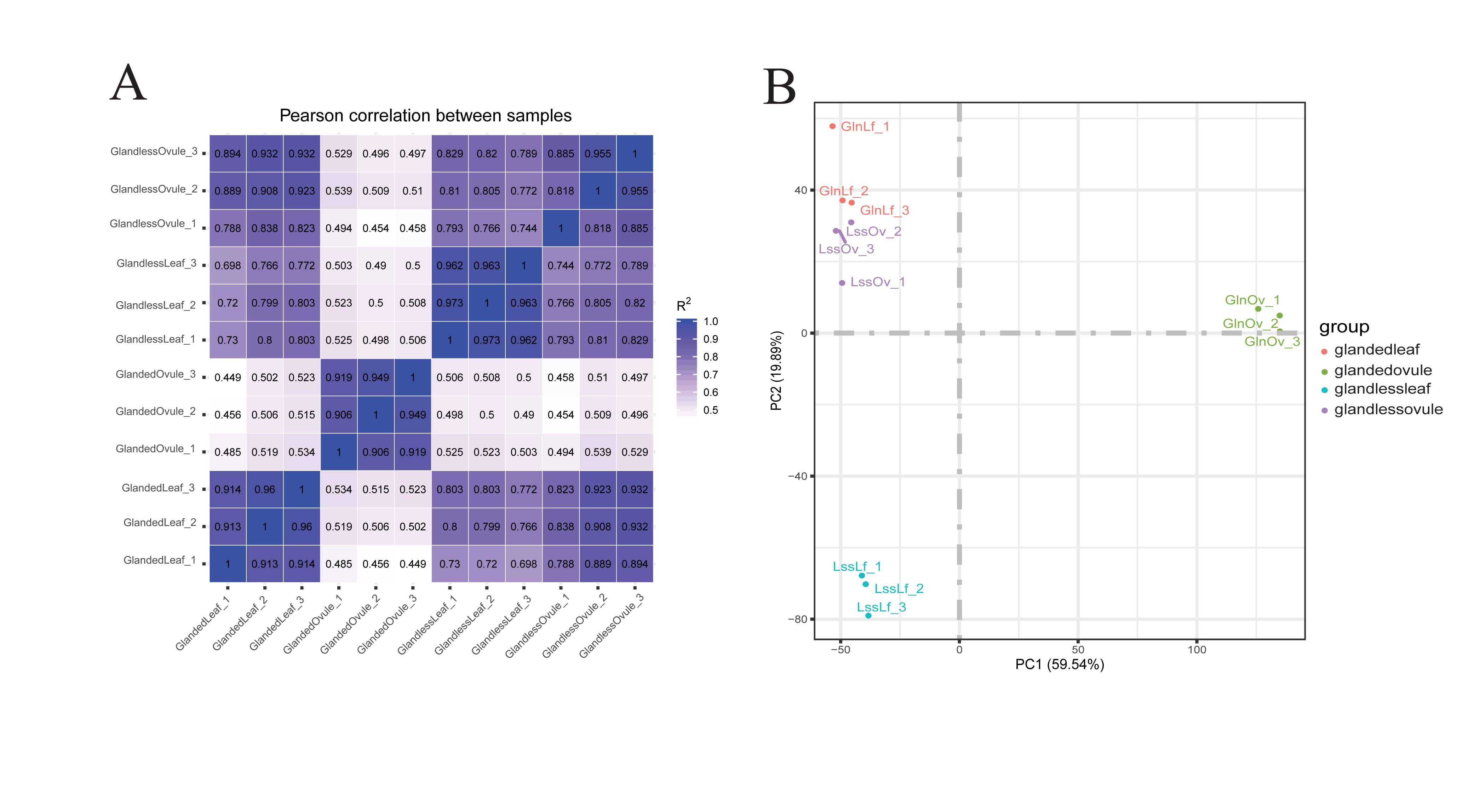

Supplement: Supplementary file 1 [file genes-13-01452-s001.zip › figures/Figure S1_Correlation_analysis_of_12_samples.jpg]

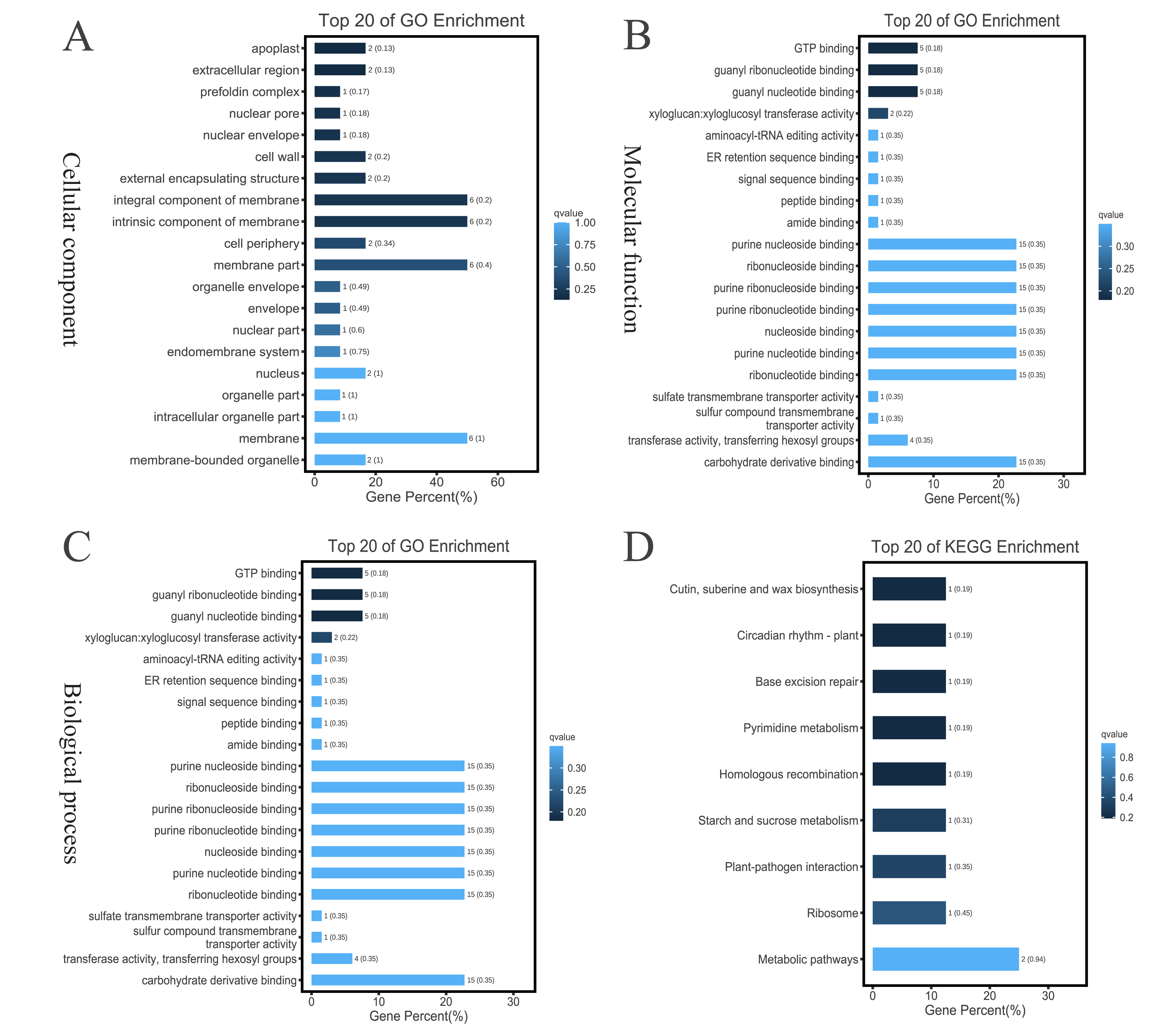

Supplement: Supplementary file 1 [file genes-13-01452-s001.zip › figures/Figure S2_enrichment_analysis_of_salmon.jpg]

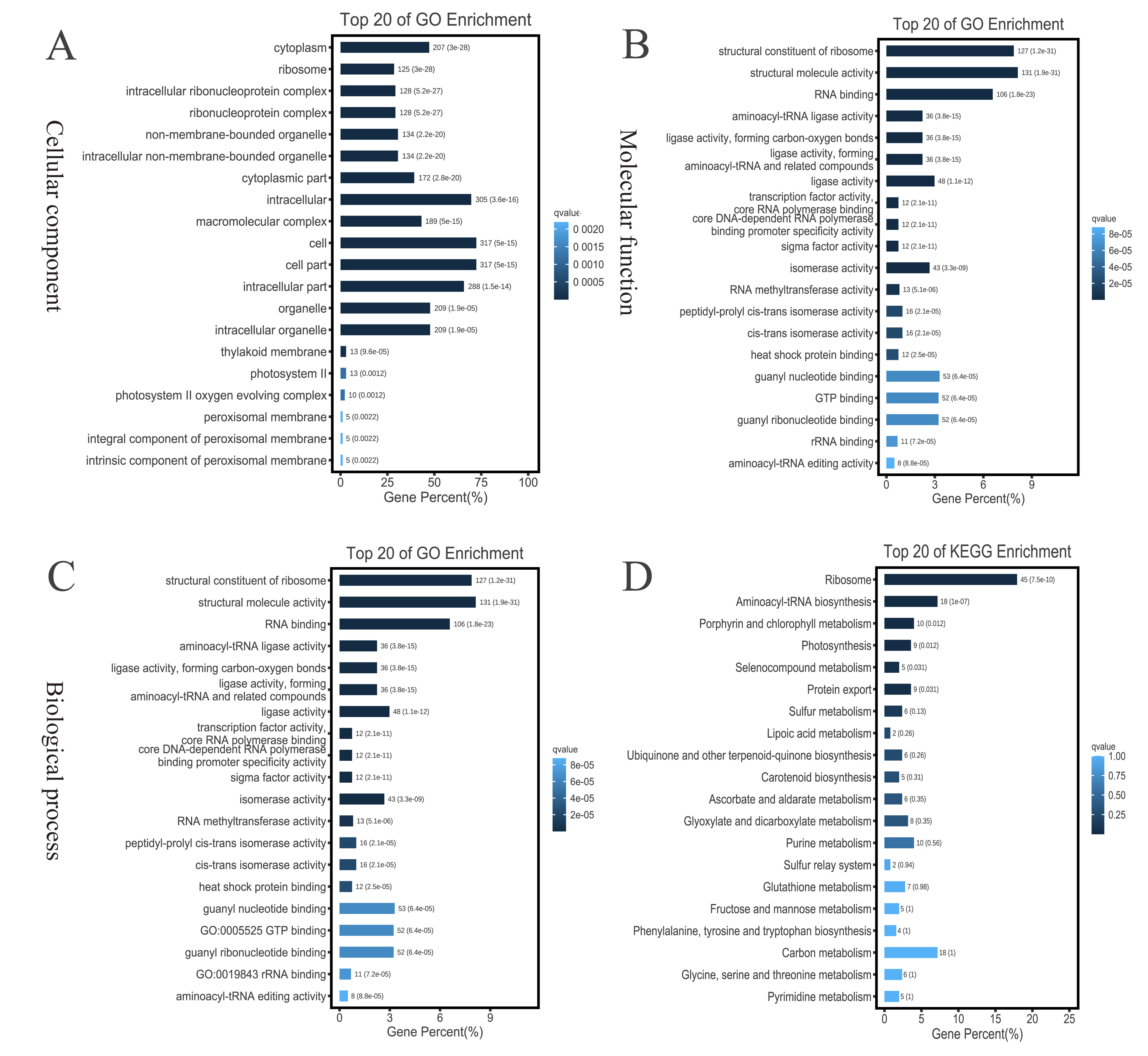

Supplement: Supplementary file 1 [file genes-13-01452-s001.zip › figures/Figure S3_enrichment_analysis_of_blue.jpg]

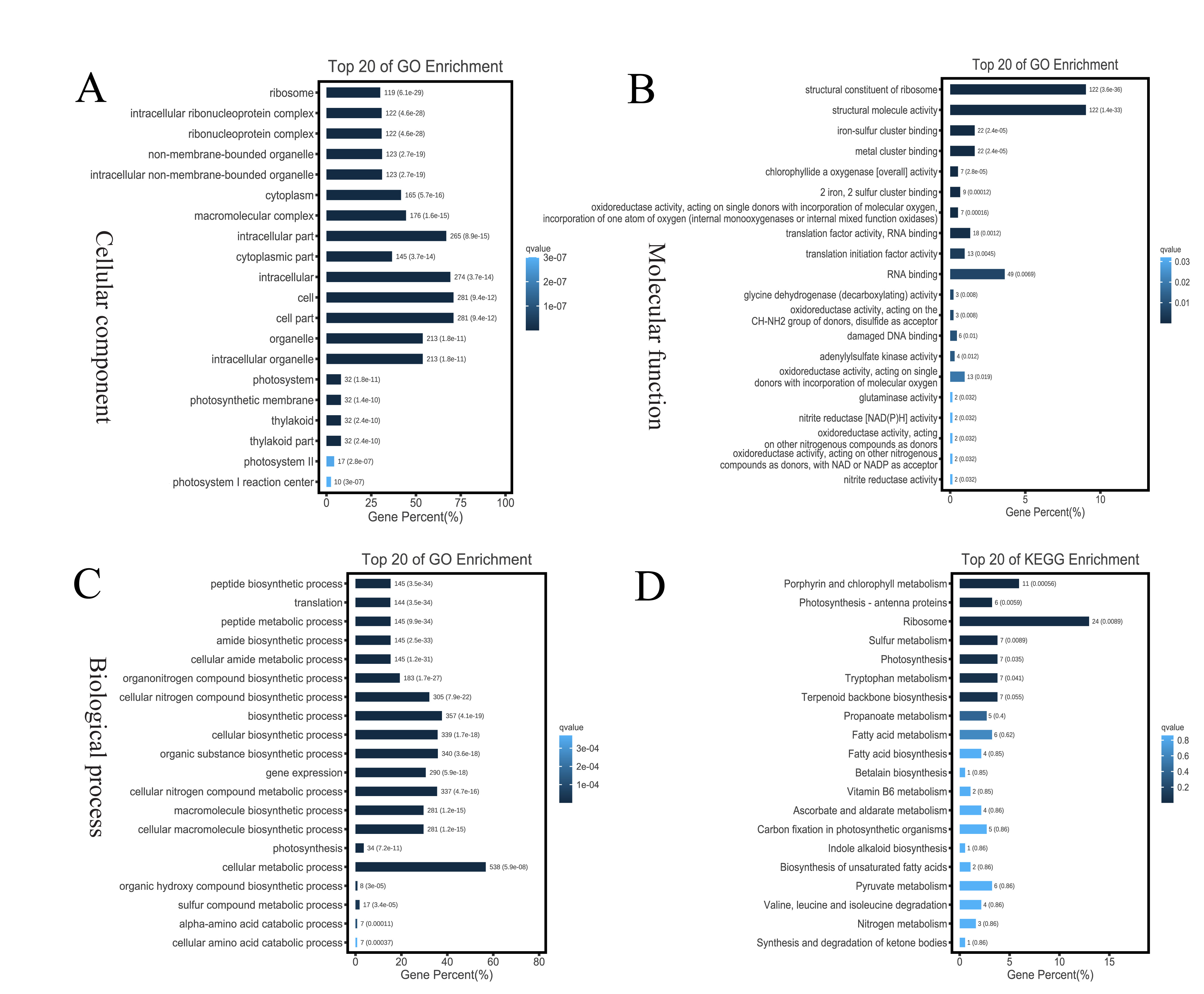

Supplement: Supplementary file 1 [file genes-13-01452-s001.zip › figures/Figure S4_enrichment_analysis_of_brown.jpg]

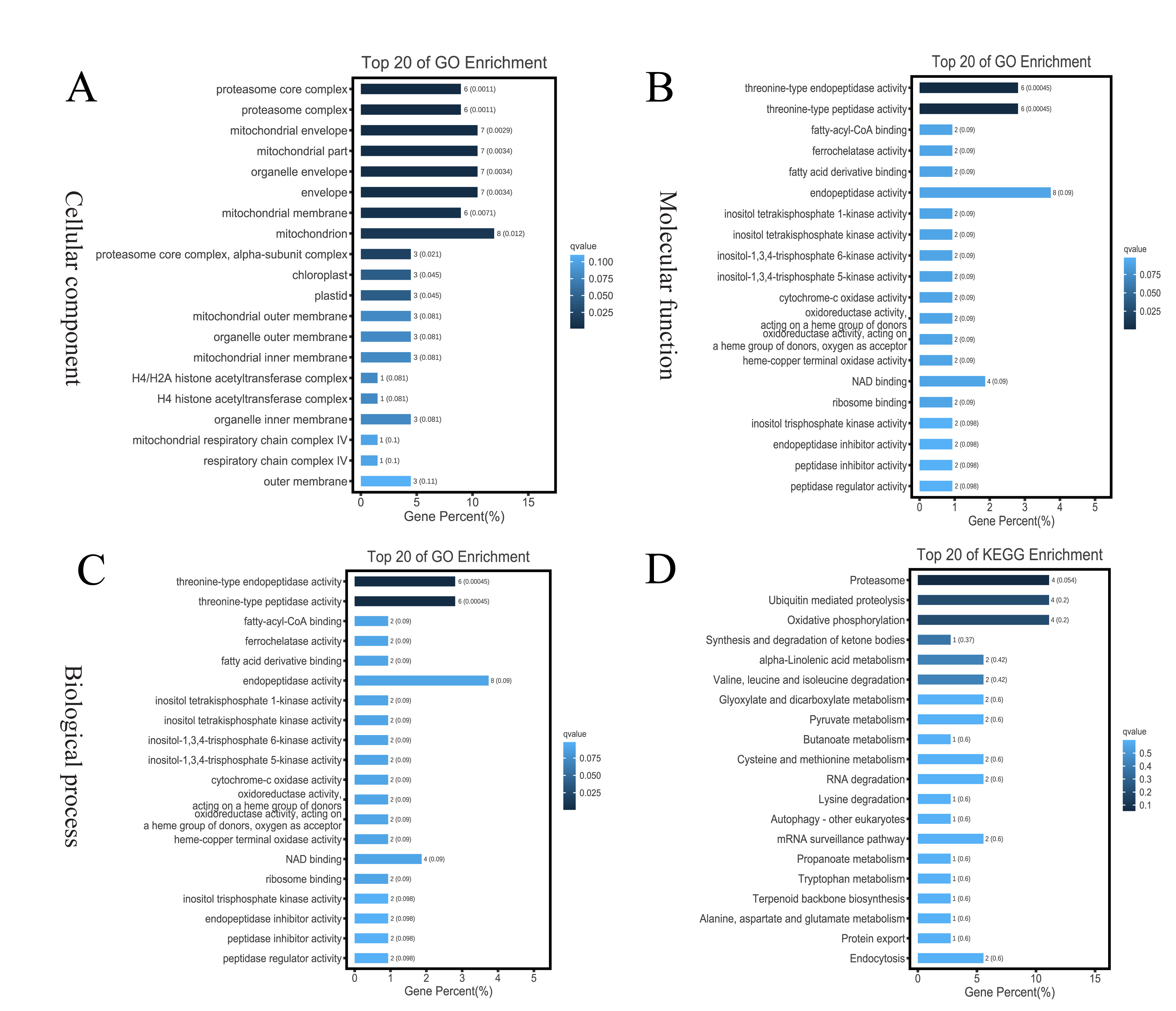

Supplement: Supplementary file 1 [file genes-13-01452-s001.zip › figures/Figure S5_enrichment_analysis_of_magenta.jpg]
